# Antigen-specific T cell responses in autoimmune diabetes

**DOI:** 10.3389/fimmu.2024.1440045

**Published:** 2024-08-15

**Authors:** Alexander J. Dwyer, Zachary R. Shaheen, Brian T. Fife

**Affiliations:** ^1^ Center for Immunology, Department of Medicine, Division of Rheumatic and Autoimmune Diseases, University of Minnesota Medical School, Minneapolis, MN, United States; ^2^ Center for Immunology, Department of Pediatrics, Pediatric Rheumatology, Allergy, & Immunology, University of Minnesota Medical School, Minneapolis, MN, United States

**Keywords:** autoimmune diabetes, autoantigens, T cells, hybrid insulin peptides, antigen specific therapy

## Abstract

Autoimmune diabetes is a disease characterized by the selective destruction of insulin-secreting β-cells of the endocrine pancreas by islet-reactive T cells. Autoimmune disease requires a complex interplay between host genetic factors and environmental triggers that promote the activation of such antigen-specific T lymphocyte responses. Given the critical involvement of self-reactive T lymphocyte in diabetes pathogenesis, understanding how these T lymphocyte populations contribute to disease is essential to develop targeted therapeutics. To this end, several key antigenic T lymphocyte epitopes have been identified and studied to understand their contributions to disease with the aim of developing effective treatment approaches for translation to the clinical setting. In this review, we discuss the role of pathogenic islet-specific T lymphocyte responses in autoimmune diabetes, the mechanisms and cell types governing autoantigen presentation, and therapeutic strategies targeting such T lymphocyte responses for the amelioration of disease.

## 1 Introduction

**Figure 1:**
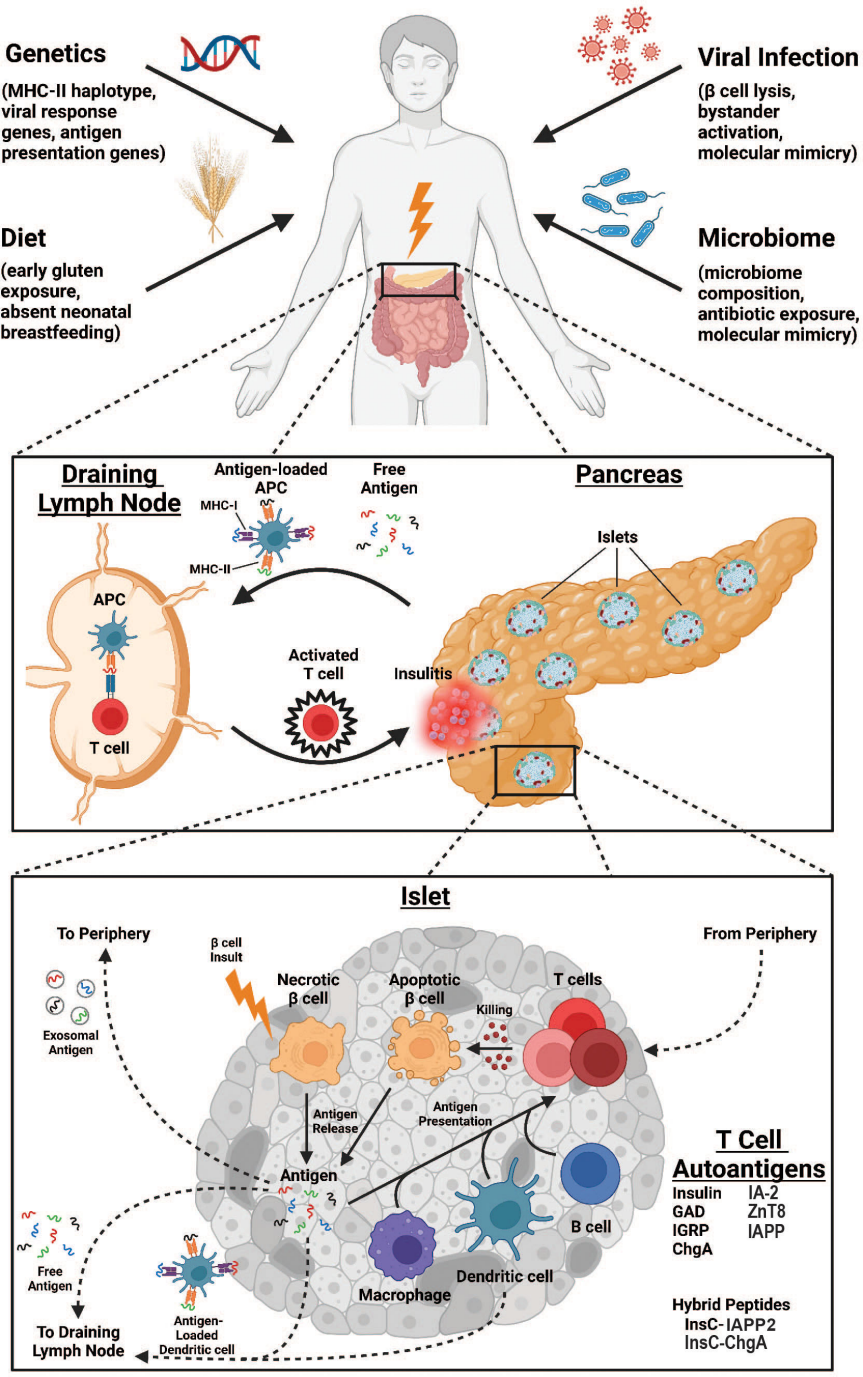
Overview of immunologic processes leading to enhanced antigen-specific T cell responses in autoimmune diabetes. Environmental factors such as genetic predisposition, viral infections, dietary exposures, and microbiome composition are all risk factors for AD development (top). At the organ level, destruction of pancreatic islets releases self-antigen that drains to pancreatic lymph nodes either as free antigen or loaded to antigen presenting cells (APCs) where it is presented to antigen-specific T cells that subsequently traffic to pancreatic islets to propagate further damage (middle). At the islet level, an unknown insult induces βcell necrosis and antigen release, initiating several pathways to T cell antigen presentation. Antigen may be drained to local lymph nodes either as free antigen or loaded to dendritic cells as above. Alternatively, antigen may enter the vasculature as exosomes for antigen presentation peripherally. Finally, T cell antigen presentation can occur within the islet, mediated by macrophages, dendritic cells, or B cells. Ultimately, T cells enter the islet proper from the periphery to promote killing of βcells through apoptotic mechanisms, further increasing the pool of available self-antigen.

Autoimmune diabetes is characterized by the destruction of the insulin-producing β-cells within the pancreatic islets of Langerhans. Although the events preceding the onset of disease are not entirely understood, many putative precipitating risk factors have been investigated. A complex interplay among risk factors including host genetic susceptibility, environmental exposures (particularly viral infection), gastrointestinal microbiome composition, and dietary contributions lead to tissue destruction in a manner principally driven by T cells (summarized in **Figure 1**). This review will focus on the major β-cell antigens and associated autoreactive T lymphocyte populations that are responsible for mediating diabetes pathogenesis. This review will focus primarily on mechanisms of diabetes pathogenesis elucidated in the non-obese diabetic (NOD) mouse, henceforth referred to as autoimmune diabetes, and will correlate these findings to human diabetes when possible, denoted as type 1 diabetes (T1D).

### 1.1 Genetic risk

Extensive epidemiologic and genetic studies have demonstrated that certain genetic variations afford significant risk to the development of T1D. The overall risk for developing T1D is estimated around 0.4%, and this risk increases to approximately 6% in children of diabetic parents, demonstrating a heritable genetic component to T1D risk (1). Per genome-wide association studies (GWAS), over 50 distinct genetic loci have been identified in humans that are associated with T1D (2, 3). Generally, these susceptibility genes may be approximately grouped by the gene product’s characterized role in coordinating anti-viral responses, autoantigen formation or presentation, or T cell signaling responses, and have been discussed in detail elsewhere (1, 4).

Of these gene products, the human leukocyte antigen (HLA) region on chromosome 6p21 represents the strongest genetic association for inherited risk of T1D development, as elucidated by both GWAS and linkage studies (1–3). HLA regions correspond to major histocompatibility complex (MHC) molecules, which are cell surface receptors that form complexes with principally either endogenous peptides (class I) or extracellular, phagocytosed peptides (class II) that are then displayed to T cells for recognition. T cell receptor (TCR) binding to these MHC:peptide complexes is essential for induction of central tolerance (via deletion of auto-reactive T cells in the thymus), continuous maintenance of peripheral tolerance (via suppression of autoreactive T cells that escaped thymic deletion), and for appropriate activation when foreign peptides are detected during infection and cancers [reviewed in (5)]. The highest risk HLA haplotypes are MHC class-II molecules colloquially termed “DR4-DQ8,” specifically DR4 (DRB1 allele)-DQA1*03:01-DQB1*03:02 (4). These high-risk haplotypes are thought to confer diabetes risk through different amino acid residues in the MHC peptide binding pocket which alters both the register in which peptides bind to MHC and the binding affinity of the MHC:peptide complex when presented to T cells.

Genetic and environmental factors play a key role in susceptibility to T1D. Importantly, genetic risk alone does not account for T1D. Indeed, in surveillance studies that examined the risk of discordant monozygotic twins to both develop diabetes (e.g. long-term follow-up of a patient whose identical twin was diagnosed with T1D), the risk of identical twins to both develop diabetes is estimated to be 39–65% (6–10). Therefore, there are likely additional environmental factors that confer susceptibility to diabetes development.

### 1.2 Viral infection

Viral infections are hypothesized to be both environmental triggers and accelerators of T1D pathogenesis [reviewed in (11–13)] and are well described contributors to initial β-cell damage and the initiation of a selective, T cell dependent autoimmune response. In humans, several viruses have been associated with the development of T1D by serological methods, of which coxsackieviruses and other enteroviruses are the most well described (11–14). Notably, enteroviral RNA and capsid proteins have been detected in the pancreatic islets of living patients with new autoimmune diabetes diagnosis (15). Coxsackievirus has also been isolated from the pancreas of a deceased patient following new-onset diabetic ketoacidosis.

Coxsackievirus virus strain (coxsackievirus B4 – CVB4) isolated from new-onset diabetic patients has also been shown to stimulate the rapid development of diabetes in inoculated mice (16), demonstrating a causative effect of acute viral infections with the triggering of β-cell autoimmunity. Since, viral infections in mouse and rat strains have since been extensively used to better elucidate the underlying mechanisms governing β-cell destruction and the onset of autoimmune diabetes, including coxsackievirus infection in the NOD mouse (17), encephalomyocarditis virus (EMCV) in certain susceptible mouse strains (18, 19), and Kilham’s Rat Virus (KRV) in the diabetes-resistant BB-rat (20). There are multiple mechanisms by which viruses may lead to the development and acceleration of pancreatic β-cell autoimmunity, including direct infection of β-cells (leading to cell lysis and antigen release in addition to direct loss of β-cell mass), bystander activation (β-cell destruction via a robust inflammatory cytokine response), and molecular mimicry (viral antigens which share sufficient homology with autoantigens to stimulate an autoimmune response) (11–13, 21, 22). Together, these mechanisms may contribute to diabetes initiation through the induction of an inflammatory environment and enhanced autoantigen presentation.

### 1.3 Gastrointestinal microbiome and diet

Gastrointestinal homeostasis is critical for proper immune system function, and derangements of the intestinal microbiome and diet may afford increased risk for T1D development (23). Several studies have demonstrated that administration of antibiotics or probiotic bacterial cocktails to diabetes-susceptible mice are capable of altering gastrointestinal microbiome diversity with a concomitant decrease in autoimmune diabetes incidence, suggesting that various bacterial species differentially contribute to influencing self-tolerance (24–27). Supporting this notion, principle component analysis of autoantibody-positive prediabetic patients have fewer butyrate- and lactate-producing bacteria such as bifidobacterium, which are hypothesized to reduce gut permeability and inflammation, and an increased proportion of *Bacteroides* species (4.3% compared to 2.0% autoantibody negative controls) (28), and the diversity of these gastrointestinal bacteria changes throughout time in seroconverter and diabetic children (28–32). Further, neonates born by Caesarean section delivery have an altered microbiome, and these infants have a 20% increased rate of T1D (33, 34). However, this relationship may not be causal, as diabetes-susceptible mice born by Caesarean section have no change in diabetes incidence, despite having increased *Bacteroides* and *Lachnospiraceae* species and fewer T regulatory cells (T_regs_) as adults (35). Importantly, microbiome-specific T cell responses are thought to be induced by a combination of mechanisms, including molecular mimicry [e.g. magnesium transporter protein (Mgt)_267-275_ stimulation of islet-specific CD8^+^ T cell responses (36)] and diminished T_reg_ quantity (37, 38). Given these observations, we posit that simultaneous stimulation of autoreactive effector T cells via molecular mimicry and decreased T_reg_-mediated suppression results in enhanced autoimmunity.

Diet is thought to influence both gastrointestinal homeostasis and microbiome composition in the context of T1D development. Neonatal feeding method is one component of diet thought to influence T1D with evidence suggesting that any amount of breastfeeding in the neonatal and infant periods decreases the risk for T1D development (39, 40). Further, a link between dietary gluten consumption and T1D has been suggested given that diabetes-susceptible mice with reduced or absent wheat and barley proteins in their diets develop delayed diabetes onset, and in humans approximately 1.7–16.4% of patients with T1D also have celiac disease, an autoimmune disease that causes immune intolerance to gluten (41, 42). In humans, this association is more complex given that effects on autoantibody levels and disease state depend upon the age at which exposure occurs (43–47).

### 1.4 The non-obese diabetic mouse

The NOD mouse was derived from Cataract Shionogi (CTS) mice in 1980, and identified incidentally in a female mouse exhibiting polyuria, glucosuria, rapid weight loss, and lymphocytic infiltration of pancreatic islets (48). Approximately 60–80% of female and 20–30% of male mice become spontaneously diabetic within 12–14 weeks of life when maintained in germ-free environments (49–51). Interestingly, this rate of disease progression is much lower when maintained in dirty animal housing facilities (49), and viral infection in the NOD mouse may either confer protection from or accelerate diabetes onset depending on the age of the mouse (17), suggesting that the timing of infection and local cellular environment may dictate whether infection provokes an autoimmune response or whether peripheral tolerance to autoantigens are maintained.

The NOD mouse has remained a dominant animal model for the study of autoimmune diabetes over the last four decades given that it shares a number of similarities with human diabetes [reviewed in (50, 51)]. First, numerous autoantigens have been identified in both humans and the NOD mouse, including insulin, glutamic acid decarboxylase (GAD65), islet-specific glucose-6-phosphatase catalytic subunit-related protein (IGRP), insulinoma antigen-2 (IA-2), phogrin (IA-2β), chromogranin A (ChgA), islet amyloid polypeptide (IAPP), and islet zinc transporter (ZnT8) (52–60). Second, the genetic susceptibility of the NOD mouse mirrors that of humans such that MHC-II molecules (termed I-A^g7^ in the NOD mouse; DQ8 in human) afford the highest risk for associated disease (1, 4, 61), which we posit allows for insufficient central tolerance to key pathogenic peptides. Functionally, human DQ8 and mouse I-A^g7^ have been shown to share similar peptide binding and registers (62) and replacing transgenic mice with DQ8 maintains islet reactivity and diabetes incidence (63), highlighting the central role of antigen presentation in autoimmune disease risk. There are additionally over 40 other non-MHC genes associated with diabetes risk that are involved in anti-viral responses, auto-antigen formation or presentation, or T cell signaling responses (1, 4). Third, self-antigen-reactive CD4^+^ T cells are essential for both early and late stages of disease development (50, 51, 64, 65). Additionally, macrophages, dendritic cells, CD8^+^ T cells, and B cells have all also been identified infiltrating islets in humans and mice and have characterized roles with disease initiation and acceleration (50, 51). In line with these similarities, the study of NOD mice has revealed many mechanistic insights into T1D disease pathology; the focus of this review will be to discuss antigenic targets in the NOD mouse as well as the cellular mechanisms by which presentation of these antigens to T cells result in the loss of tolerance and induction of autoimmune diabetes.

## 2 Antigenic targets of T cells

### 2.1 Insulin

Although many β-cell protein self-antigens are known targets in T1D (summarized in **Figure 1**), several are thought to be more pathogenic than others and will be discussed here. Among these, insulin is perhaps the most well-known. Specifically, the InsB_9-23_ epitope is likely the critical antigenic portion of insulin given the large number of islet-infiltrating T cell clones that are reactive to InsB_9-23_ peptide (66–68). Supporting these data, it was subsequently found that NOD.*ins1^-/-^ins2^-/-^* mice expressing a transgene encoding hormonally functional insulin but with a tyrosine-to-alanine point mutation at position 16 of the insulin B-chain (NOD.Y16A mice) prevents I-A^g7^ binding and antigen presentation and as a result, these mice do not develop autoimmune diabetes (53, 69, 70). Interestingly, transfer of splenocytes from NOD.Y16A mice to NOD.*scid* recipients induces disease but at a rate slower than splenocyte transfer from NOD mice, suggesting that InsB_9-23_-specific T cells may participate in disease initiation but require a latency period of several weeks to recruit participation of other islet antigen-specific responses (53).

Both murine I-A^g7^ and human DQ8 MHC-II molecules bind peptide for antigen presentation using a core of nine amino acids, meaning that the fifteen amino acid InsB_9-23_ peptide could interact with MHC-II in multiple different positions, termed “registers” (71, 72). Of the nine amino acids comprising core binding to I-A^g7^ and DQ8, binding of a negatively charged residue at position 9 (P9) is most important for determining stability of the peptide:MHC-II interaction (62). This is due to a polymorphism at position 57 of the β-chain of all HLA-II diabetes susceptibility alleles where a negatively charged aspartic acid residue is replaced by a neutral amino acid, leaving an exposed positively charged surface that alters the P9 pocket binding parameters (72, 73). The P9 pocket is thought to be important in determining the binding affinity of different insulin peptide registers to murine and human MHC-II (74).

Three insulin binding registers have been studied most frequently: InsB_12-20_ (register 1), InsB_13-21_ (register 2) and InsB_14-22_ (register 3). Registers 1 and 2 bind weakly to I-A^g7^, with register 2 satisfying the P9 position negative charge condition through its glutamic acid while register 1 has a neutral glycine at this position (74). It has been suggested that I-A^g7^ and other β57 polymorphic MHC-II molecules select for TCRs with aspartic acid and glutamic acid residues within the CDR3β region, allowing neutralization of the positively charged MHC-II surface following binding of peptides with neutral amino acids at P9 in a process termed the “P9 switch” (75, 76). This mechanism would explain how register 1 can bind I-A^g7^ with low affinity to allow InsB_12-20_-specific CD4^+^ T cells to escape negative selection and home to pancreatic islets (76). Using InsB_12-20_-TCR specific transgenic 8F10 CD4^+^ T cells, it has been shown that these populations encounter insulin antigen directly in pancreatic islets without a need for initial antigen presentation at 4 weeks in the draining pancreatic lymph nodes (pLN) (77). Importantly, CD4^+^ T cells specific for register 1 are thought to recognize antigen only when antigen presenting cells (APCs) uptake InsB_9-23_ peptide and subsequently expand in the periphery (78). In contrast, CD4^+^ T cells specific for register 2 recognize antigen after APC uptake of full-length insulin and are deleted in the thymus (78). These studies suggest that development of autoreactive T-lymphocytes to insulin occurs in a manner dependent on recognition of InsB_9-23_ in register 1 but not 2; indeed, register 1-specific pLN-derived CD4^+^ T cell clones transfer disease to NOD.*scid* recipients (78, 79).

Insulin register 3 binds poorly to I-A^g7^ due to a positively charged arginine residue occupying the P9 position, yet despite this, several insulin-specific CD4^+^ T cell clones have been found to recognize InsB_9-23_ bound in register 3 but not registers 1 or 2 (56). Further, the majority of InsB_9-23_-specific CD4^+^ T cells in NOD mice target register 3 with an arginine to glutamic acid mutation at P9 of the insulin chain (R22E) which enhances binding stability and segregates into two distinct populations: those with responses *inhibited* by additionally mutating B:21 glutamic acid to glycine (“P8E”) and those with *augmented* responses (“P8G”) (80–82). Interestingly, R22E register 3-specific PBMCs are also found in human patients with T1D (83). Additionally, agonistic R22E register 3 insulin peptide converts naïve CD4^+^ T cells into FoxP3^+^ T_regs_ and prevents autoimmune diabetes in NOD mice, supporting a crucial role for register 3 binding MHC-II in stimulating diabetogenesis (84). Similarly, NOD mice treated with a monoclonal antibody targeting R22E register 3 in the context of I-A^g7^ delays autoimmune diabetes (85). Although the mechanism underlying natural formation of the P8E and P8G insulin mimotopes is unknown, a possible candidate may be ligation of InsB_14-20_ to a separate unidentified peptide comprising the relevant amino acids necessary to bind P8 and P9 to form a hybrid peptide neoantigen (see below). These data together suggest that presentation of InsB_12-23_ peptide via diabetes susceptibility MHC-II alleles can occur through insulin binding in three registers, but further investigation is required to determine whether T cell responses directed against one register dominate over the others.

### 2.2 Glutamic acid decarboxylase

Glutamic acid decarboxylase (GAD) is a β-cell self-antigen expressed as the GAD65 and GAD67 isoforms in human and murine islets respectively (86, 87). In contrast to insulin, no single critically targeted GAD epitope has been identified, and contributions of GAD-specific T cell responses to disease are not well-understood. Many newly diagnosed patients produce GAD65-specific antibodies, and these antibodies can be used to predict T1D disease status with marginal specificity and positive predictive value (88–90). Additionally, GAD65_555-565_-reactive CD4^+^ T cells can be found in the peripheral blood of diabetic patients, and peripheral GAD65-reactive T cells are known to mount T_H_1-like IFNγ secretion responses (91, 92). In NOD mice, GAD suppression prevents disease, suggesting that GAD-specific responses are required for autoimmune diabetes progression (93). A large number of GAD65-specific T cell hybridomas recognize GAD65_206-220_, likely due in part to an acidic glutamic acid residue occupying the positively charged P9 pocket of I-A^g7^, but GAD65_206-220_-specific T cells have not been shown to be pathogenic (72, 94, 95). IFNγ secretion by GAD65_509-528_- and GAD65_524-543_-specific T cells is observed by 4 weeks in NOD mice, and analogous responses by coxsackievirus mimotope GAD65_246-266_-specific T cells emerge by 7 weeks (96). The GAD65_524-543_ peptide in particular has been further implicated as a pathogenic epitope given that adoptive transfer of a T_H_1-like GAD65_524-543_-specific CD4^+^ T cell clone induces diabetes in NOD.*scid* mice (97).

In spite of this evidence, GAD65_524-543_ T cell proliferative responses are also observed in the diabetes-resistant H-2^g7^-restricted B10.*H-2^g7^* and NOD.B6*^Il2-Tshb^* mouse strains, suggesting that GAD_524-543_ reactive T cells alone are insufficient to stimulate autoimmune diabetes (98). Further, T_H_1-like CD4^+^ T cell clones targeting murine GAD65_524-543_ or human GAD65_247-266_ could not accelerate disease in NOD mice or cause disease in NOD.*scid* mice, though this may be due to a low-affinity TCR on generated hybridomas given successful disease induction in other settings (99). Additionally, a GAD65_515-524_-specific CD8^+^ T cell clone was unable to induce disease despite secreting large amounts of IFNγ and accelerating insulitis upon transfer to NOD mice (100). Although these studies do not collectively clearly explain the role of antigen-specific T cell responses in autoimmune diabetes it is likely that GAD65-specific T cell responses evolve over time and synergize with other antigen-specific T cell populations to orchestrate destruction of pancreatic β-cells.

### 2.3 Insulinoma antigen

Receptor-type tyrosine-protein phosphatase-like N, also known as insulinoma antigen-2 (IA-2) and IA-2β (also termed phogrin or ICA512) are transmembrane proteins expressed on the secretory granules of neuroendocrine cells, including pancreatic islets and neurons. IA-2 was first identified as an autoantigen by analyzing sera from patients with T1D, in which the autoantibodies preferentially bound to the intracellular domain of IA-2 (101, 102). The presence of IA-2 autoantibodies, along with insulin and GAD, strongly correlated with the incidence and rapidity of type 1 diabetes onset (103). Three observations in human patients suggest that IA-2, similar to GAD and ZnT8 (discussed next), may be pathogenic but not an early, primary initiating antigen of autoimmunity. First, T cells isolated from patients were more likely to be responsive to IA-2 antigen (104), demonstrating their pathogenic potential. Second, while the presence of both IA-2 and GAD antibodies in childhood-onset T1D were associated with MHC-II, this association was with the DRB1 and DQB1 alleles, respectively, and not highest risk allele DR4-DQ8 (105). Third, IA-2 and GAD antibodies are more likely to appear after anti-insulin antibodies and correlate with an older age of T1D diagnosis (105, 106), suggesting that their appearance may occur during epitope spreading and disease progression rather than the primary insult that triggers autoimmunity to β-cells.

### 2.4 Zinc transporter ZnT8

The Zinc Transporter ZnT8 is an islet-specific protein located on the membrane of insulin secretory granules, that mediates enrichment of zinc within the granules to store insulin as tightly packaged hexamers (107). Additionally, ZnT8 is detectable on cell surface of β-cell cultures following glucose-stimulated insulin secretion (108), providing a mechanism by which this islet-specific antigen is accessible to generate an immunologic response. ZnT8 was first identified as an associated autoantigen in human T1D by microarray expression profiling of human and rodent islet cells (109). Autoantibodies to ZnT8 are detected in 60–80% of new onset diabetics as compared to <2% of healthy controls (109). More recent studies show that the presence of ZnT8 autoantibodies portends a higher risk of diabetes independent of other islet cell autoantibodies (110), and when combined with assessment of other islet-specific autoantibodies, dramatically improves the risk of progression to diabetes (103) and autoimmunity detection rates at diabetes onset (109). These studies demonstrate clinical utility in screening for autoantibodies to ZnT8.

Given that ZnT8 autoantibodies are rarely identified in isolation and instead are more likely to be present in patients just before or at diagnosis of T1D (109), this suggests that ZnT8 autoreactivity may occur during epitope spreading and participate in acceleration or disease onset, rather than initiation of autoimmunity. Inconsistent and variable roles for both CD4^+^ and CD8^+^ T cells reactive to ZnT8 favor this observation. Isolated CD4^+^ T-cells from T1D patients exhibit significantly higher reactivity as measured by cytokine release when using a library of ZnT8-derived peptides (111). However, genome wide association studies have failed to identify consistent MHC-II genetic associations with ZnT8 antibody positivity (112). Rather, MHC class I regions, specifically HLA-A2, were associated with positive ZnT8 antibodies in patients with T1D (112, 113), and these CD8^+^ T cells are preferentially found in the pancreas of T1D patients compared to controls (113). Additionally, ZnT8 (_186-194_)-reactive CD8^+^ T cell clonotypes were found to also recognize a *Bacteroides* mimotope (113), providing an environmental link to the acceleration of autoimmunity. Limited studies in NOD mice also corroborate the hypothesis that ZnT8 participates during epitope spreading and not disease initiation. CD4^+^ T cells reactive to ZnT8 are unable to stimulate disease during transfer into NOD or NOD.*Rag*1^-/-^ mice, and are only found in the pancreas or accelerate diabetes if significant islet damage is already present (114).

While detection of insulin, GAD, IA-2, and ZnT8 antibodies have provided immense benefit in the immunological surveillance of autoimmune diabetes risk and in formal diagnosis following symptom onset, these antigens were all first detected by identification of autoantibodies in T1D patients. Given that the pathogenesis of autoimmune diabetes is driven by autoreactive T cells, there are likely to be many yet unidentified autoantigens that play fundamental roles in the initiation of autoimmunity that lack recognition by islet autoantibodies or have titers well below detection levels. In this context, work in animal models have afforded an alternative screening method, by identifying the molecular targets of diabetogenic T cell clones:

### 2.5 Islet-specific glucose-6-phosphatase catalytic subunit-related protein

Islet-specific glucose-6-phosphatase catalytic subunit-related protein (IGRP) is another β-cell self-antigen that was identified as the source of the peptide ligand responsible for activating the diabetogenic NY8.3 CD8^+^ T cell clone (115). The NY8.3 clone was isolated from the islets of a diabetic NOD mouse and was found to induce disease upon adoptive transfer of IGRP-specific CD4^+^ T cells (116, 117). It was further discovered that this clone used a TCRα CDR3 sequence that was similar to sequences used by many islet-infiltrating β-cell-specific CD8^+^ T cells in NOD mice, suggesting that the NY8.3 antigen is a common target of cytotoxic CD8^+^ T cells in murine autoimmune diabetes (118, 119). For these reasons, the NOD.NY8.3 mouse was developed through introduction of transgenes encoding K^d^-restricted TCR sequences derived from the NY8.3 clone and found to develop accelerated diabetes relative to littermate non-transgenic controls (120). When similar mice are depleted of macrophages, disease is entirely prevented and T_H_1-skewing of CD4^+^ T cells is impaired, demonstrating a requirement of macrophages for activation of CD8^+^ T cells in NOD.NY8.3 mice (121). Using high-performance liquid chromatography on cellular extracts from the pancreatic β-cell line NIT-1, fractions corresponding to IGRP_206-214_ were discovered to specifically stimulate NY8.3 cells, and tetramer staining identified endogenous IGRP_206-214_-specific CD8^+^ T cells in the islets and blood of NOD mice (115, 122). These experiments confirmed IGRP_206-214_ as the antigen of diabetogenic NY8.3 CD8^+^ T cells.

Following identification of IGRP_206-214_ as the NY8.3 CD8^+^ T cell antigen, several studies in NOD mice and humans have furthered knowledge regarding the pathogenicity of this epitope. Interestingly, complete tolerance to IGRP does not prevent disease in NOD mice despite elimination of IGRP_206-214_-specific CD8^+^ T cells (122). However, when depletion of IGRP_206-214_-specific CD8^+^ T cells occurs in a manner that spares low-avidity clones, disease in NOD mice is prevented. These data suggest that low-avidity IGRP_206-214_-specific clones are non-pathogenic and actively suppress disease in a manner dependent on suppression of autoreactive T cell clones that target additional self-peptides beyond IGRP itself (123). T cell IGRP antigen is encountered selectively in the pLN, because CFSE-labeled NY8.3 CD8^+^ T cells proliferate in the pLN but not the inguinal lymph node (iLN) following adoptive transfer to NOD mice (122). Interestingly, peripheral IGRP_206-214_-specific CD8^+^ T cell cytotoxicity can be used to predict disease, as NOD mice with splenocyte effector activity above a certain threshold develop disease while those without effector activity do not (124). When transgenic NOD mice express human HLA-A*0201 in place of murine MHC-I, islet-infiltrating CD8^+^ T cells were found to respond to IGRP_228-236_, IGRP_265-273_ and IGRP_337-345_, implicating these epitopes as potentially relevant to human disease (125). Of these epitopes, IGRP_265-273_ is a likely target of antigen-specific CD8^+^ T cell responses in patients with T1D because it is entirely conserved between mice and humans (126).

Supporting a role for the pathogenic potential of the IGRP_265-273_ epitope in humans, *in vitro* expanded IGRP_265-273_-specific CD8^+^ T cells derived from blood of a diabetic patient effectively lyse target cells in a peptide-specific manner (127). Further, IGRP_265-273_-specific CD8^+^ T cells utilize shared TCRα-chains across multiple patients (127). Despite this, IGRP_265-273_-specific CD8^+^ T cells from diabetic patients are incapable of producing IFNγ, suggesting an inability of these cells to amplify their own inflammatory response (126, 127). This lack of IFNγ secretion is likely epitope-specific, as peripheral CD8^+^ T cell IFNγ responses are observed against IGRP_215-223_ and IGRP_222-230_, two peptides that were computationally predicted to bind well to HLA-A*0201 (126). Detection of peripheral CD8^+^ T cell responses directed against IGRP_265-273_ and IGRP_228-236_ is also observed in recent-onset diabetic patients but not healthy controls (128). Importantly, IGRP-specific T cell responses appear to be most pathogenic when mediated by CD8^+^ T cells, as CD4^+^ T cells specific for a number of IGRP epitopes exist in similar proportions in the periphery of diabetic and healthy subjects and produce similar amounts of IFNγ and IL-10 (129). Altogether, these data support pathogenic contributions to autoimmune diabetes development by IGRP-specific CD8^+^ T cells in both NOD mice and humans.

### 2.6 Chromogranin A

Chromogranin A (ChgA) is a neuroendocrine protein with diverse tissue expression including pancreatic β-cells where it is critical for regulating intracellular vesicle trafficking dynamics and insulin secretion (130–133). The discovery of ChgA as an T1D self-antigen is related to its ability to stimulate the diabetogenic BDC-2.5 CD4^+^ T cell clone, whose cognate antigen went unrecognized for many years (57). The BDC-2.5 clone was identified among a panel of islet-specific CD4^+^ T cell clones expanded from the pancreas of a diabetic NOD mouse and was capable of inducing rapid diabetes in prediabetic NOD mice after only 2-weeks post-adoptive transfer in a manner partially dependent upon CD8^+^ T cell cytotoxicity (134–137). A transgenic NOD mouse harboring the BDC-2.5 TCRα- and β-chains on CD4^+^ T cells (NOD.BDC-2.5) was subsequently developed and found to become diabetic with higher penetrance than non-transgenic NOD mice despite a similar age of disease onset (138). Experiments involving transfer of diabetogenic CD4^+^ T cell clones of different antigen specificities to NOD.*scid* mice demonstrated that BDC-2.5-specific cells promoted disease earlier than other clones (139). Initially, GAD65 was the suggested antigen targeted by BDC-2.5 clones given the sequence similarity between GAD65_528-539_ and several synthetic peptides stimulatory to BDC-2.5 CD4^+^ T cells (140). However, the BDC-2.5 CD4^+^ T cell proliferation induced by this peptide was low and a similar peptide (GAD_521-535_) failed to cause BDC-2.5 IFNγ secretion, so this hypothesis was quickly abandoned (140, 141). Other CD4^+^ T cells responsive to synthetic peptides with BDC-2.5 CD4^+^ T cell stimulatory capacity were investigated through tetramer analysis and found in NOD mouse islets and spleens, but these experiments similarly were not able to elucidate the BDC-2.5 antigen (142).

The first clue that ChgA is an antigenic source for BDC-2.5 CD4^+^ T cells was the observation that the isolated secretory granule fraction from subcellular fractionation of β-cells stimulated BDC-2.5 T cells (143, 144). Further experiments involving chromatographic fractionation of β-cell membrane lysate followed by mass spectrometry of fractionated peptides identified ChgA as a candidate antigen for the BDC-2.5 clone due to its presence in a series of highly stimulatory elution fractions (57). Additionally, it was shown that NOD.*ChgA^-/-^* mouse islets do not stimulate BDC-2.5 CD4^+^ T cells to produce IFNγ, and disease was completely prevented, and only slight insulitis was observed in these mice (57, 145). While these data implicated ChgA-specific autoreactive T cells in autoimmune diabetes, genetic deficiency of chromogranin also likely affects disease trajectory via other β-cell intrinsic mechanisms. Specifically, genetic deletion of ChgA in the NOD mouse may result in β-cell secretory granule deficits given the contributions of ChgA to normal vesicular trafficking (131, 132). This is supported by results showing that NOD.*ChgA^-/-^* mice produce less insulin on glucose challenge, and several insulin-specific hybridomas are less responsive to NOD.*ChgA^-/-^* islets relative to NOD islets (133).

Additional experiments further implicated ChgA as a potential source of BDC-2.5-stimulatory peptides: the high-affinity pS3 BDC-2.5 antigen mimotope was found to have sequence similarity to the ChgA cleavage product WE14, and WE14 was capable of weak BDC-2.5 CD4^+^ T cell stimulation (57). WE14-reactive CD4^+^ T cells have also been detected in diabetic patients (146). Confusingly, WE14 could not elicit the same level of IFNγ secretion from BDC-2.5 CD4^+^ T cells as β-cell membrane or the pS3 mimotope, and the N-terminus of WE14 is predicted to occupy the C-terminal half of the I-A^g7^ binding groove, leaving positions 1–4 unfilled (57). Vasostatin-1-derived ChgA_29-42_ peptide was found to stimulate BDC-2.5 proliferation better than WE14, but even this peptide did not stimulate as robustly as the pS3 mimotope (147). These data suggested that, while ChgA-derived peptides are clearly implicated in BDC-2.5 CD4^+^ T cell activation, an unknown alteration likely changes the amino acid sequence of a ChgA-derived peptide in some manner to increase its propensity for diabetogenic T cell activation. However, the details of this process and the identity of the natural BDC-2.5 antigen remained elusive.

### 2.7 Islet amyloid polypeptide

Similar to the identification of ChgA as the peptide targeted by the BDC-2.5 clone, islet amyloid polypeptide (IAPP) was identified via investigating the cognate antigen of the diabetogenic BDC-6.9 CD4^+^ T cell clone (135–137, 148). The BDC-6.9 clone was isolated from the pancreas of a diabetic NOD mouse and caused disease upon transfer to prediabetic mice after a 2-week latency period, similar to the BDC-2.5 clone (135–137). Interestingly, adoptive transfer of BDC-6.9 CD4^+^ T cells to NOD.*scid* recipients induces rapid disease while BDC-2.5 CD4^+^ T cells require co-transfer with CD8-enriched splenocytes, suggesting that the BDC-6.9 clone harbors intrinsic β-cell destructive properties independent of CD8^+^ T cell cytotoxicity in this setting (137). However, BDC-6.9 and BDC-2.5 CD4^+^ T cell clones (derived from T cell lines) transfer diabetes with equal onset and incidence to NOD mice, implying equivalent diabetogenic potential in spontaneous disease (137).

Identification of the antigenic source of the BDC-6.9 clone occurred quickly due to an early observation that transfer of BDC-6.9 CD4^+^ T cells to F_1_ progeny of NODxBALB/c breeding pairs did not cause diabetes, yet BDC-2.5 CD4^+^ T cell transfer was capable of disease induction (149). These F_1_ progeny were subsequently back-crossed to BALB/c mice (BC_1_) and patterns of BDC-6.9 islet antigen inheritance were statistically assessed (148). This demonstrated that approximately 50% of the BC_1_ mice bear the BDC-6.9 antigen, which therefore mapped to a single genetic locus given the Mendelian inheritance pattern. BC_1_ mice were then genotyped to determine linkage of a known genetic locus with the ability of islets from each mouse to stimulate BDC-6.9 CD4^+^ T cell proliferation. This process identified a telomeric region on chromosome 6 that encoded IAPP as the only exclusively islet-expressed gene strongly and therefore implicated IAPP as the antigen recognized by BDC-6.9 CD4^+^ T cells.

IAPP was further implicated as a diabetogenic antigen when NOD.*IAPP^-/-^* islets were found incapable of stimulating diabetogenic BDC-5.2.9 CD4^+^ T cell IFNγ responses, and BDC-5.2.9 T cells could not cause disease upon transfer to NOD.*IAPP^-/-^* mice (150). Further, the KS20 IAPP peptide segment could stimulate BDC-5.2.9 CD4 T cell IFNγ responses, and tetramer-specific KS20-reactive CD4^+^ T cells found in the pancreata of diabetic NOD mice could be cloned and expanded to rapidly transfer disease to prediabetic NOD recipients (59, 150). These results suggested that certain CD4^+^ T cell responses in the NOD mouse target unmodified epitopes derived from the IAPP protein. However, despite successes with IAPP stimulation of the BDC-5.2.9 clone, no group was able to demonstrate stimulation of the BDC-6.9 clone with IAPP antigen, framing an unidentified amino acid sequence modification of IAPP as the putative source of antigen for BDC-6.9 CD4^+^ T cells.

### 2.8 Hybrid peptides

In recent years, hybrid peptide-specific T cell responses associated with autoimmune diabetes pathogenesis have been described in both animal models and human samples (151–155). Given that these novel peptide targets are thought to originate in the periphery as post-translational modifications (PTMs), one might predict that hybrid peptide-specific T cells would therefore escape clonal deletion in the thymus and evade central tolerance mechanisms (156). Hybrid peptide-specific T cells that recognize their cognate antigen in an inflammatory context would then be expected to become activated and expand. This is because autoimmune regulator (AIRE)-expressing medullary thymic epithelial cells (mTECs) presumably do not express post-translationally modified peptides as tissue-restricted antigens and hybrid peptide antigens are predicted to be sufficiently limiting such that it could not be transported by APCs or solubilized to freely enter thymic tissue (157). This assumption is theoretical, and no such evidence has been identified that central tolerance is either occurring or not occurring has been published to date.

Evidence for the relevance of hybrid peptides as antigens in autoimmune diabetes initially emerged with further study of the BDC-2.5 CD4^+^ T cell clone. Because the ChgA-derived peptide sequence WE14 is only slightly stimulatory to BDC-2.5 CD4^+^ T cells, transglutamination of WE14 was explored as a possible mechanism of ChgA PTM and increased antigenicity given the known role of transglutaminase (TGase) enzymes in various autoimmune processes (158, 159). TGase treatment of WE14 was found to increase antigenicity towards BDC-2.5 CD4^+^ T cells *via* covalent crosslinking and isopeptide bond formation and TGase-treated WE14 stimulated PBCMs from certain diabetic patients better than WE14 (146, 158). However, *in vivo* identification of transglutaminated WE14 peptides was never described, so this hypothesis was soon abandoned. Given the prediction that unmodified WE14 optimally binds with its N-terminus in position 5 of the I-A^g7^ peptide binding groove leaving positions 1–4 unfilled, it was soon discovered that the addition of four amino acids to the N-terminus of WE14 corresponding to positions 1–4 of the high-affinity pS3 BDC-2.5 antigen mimotope (RLGL-WE14) robustly enhanced its ability to stimulate BDC-2.5 CD4^+^ T cell IL-2 secretion (57, 160). This peculiar finding suggested that a post-translational process may generate a fusion peptide product between WE14 and another peptide at its N-terminus as the autoantigen for the BDC-2.5 clone.

Direct evidence for hybrid peptide sequences functioning as autoantigens was provided with mass spectrometric analyses performed on antigenic β-cell extract fractions, which showed that fractions corresponding to fusion peptide sequences between the insulin C-chain (InsC) and WE14 (DLQTLAL-WSRMD; InsC-ChgA) and InsC and IAPP (DQTLAL-NAARD; InsC-IAPP) stimulate IFNγ production from BDC-2.5 and BDC-6.9 CD4^+^ T cells respectively (152, 161). These results were recapitulated with direct *in vitro* culture of BDC-2.5 and BDC-6.9 CD4^+^ T cells with InsC-ChgA and InsC-IAPP peptide respectively (152, 161). Interestingly, a second unidentified β-cell extract fraction peak was found to stimulate BDC-2.5 CD4^+^ T cells even more intensely than the InsC-ChgA peak, suggestive of BDC-2.5 CD4^+^ T cell antigenic promiscuity (152). These data offer strong support for InsC-ChgA and InsC-IAPP peptides as physiologic agonists for the BDC-2.5 and BDC-6.9 CD4^+^ TCRs.

Identification of endogenous hybrid peptide-specific T cells has been possible with tetramer-tracking technologies (152, 153, 155, 161, 162). Polyclonal tetramer-specific InsC-ChgA-, InsC-IAPP- and InsB_9-23_ mimotope-reactive CD4^+^ T cells can be found in the pLN, spleen, and pancreas of prediabetic and diabetic NOD mice, and InsC-ChgA-specific CD4^+^ T cells are the most frequent of the three in pancreatic islets (152, 153, 161). Approximately 80% of InsC-ChgA-specific CD4^+^ T cells are CD44^high^CD62L^low^ in the pLN by 3-weeks and this proportion is persistent out to 10-weeks, much higher than the 20–40% expression by InsC-IAPP- and InsB_9-23_-specific CD4^+^ T cells (153). Diabetic NOD splenocytes also produce more IFNγ when cultured with InsC-ChgA peptide than InsC-IAPP or InsB_9-23_ peptides, and a greater proportion of pancreas and spleen InsB_9-23_-specific CD4^+^ T cells are FoxP3^+^ T_regs_ than InsC-ChgA- or InsC-IAPP-specific CD4^+^ T cells. These data implicate InsC-ChgA-specific CD4^+^ T cells as potent T_H_1-like effectors whereas a larger proportion of InsB_9-23_-specific CD4^+^ T cells may adopt a suppressive T_reg_ phenotype. The phenotype of InsC-IAPP-specific CD4^+^ T cells is less clear from this study, and there were fewer cells of this specificity. However, it was also shown that the total frequency and proportion of CD44^high^CD62L^low^ InsC-ChgA- and InsC-IAPP-specific CD4^+^ T cells in peripheral blood of NOD mice increases over time with disease onset much more robustly than InsB_9-23_-specific CD4^+^ T cells, strongly implicating these two hybrid peptide-reactive populations in diabetes pathogenesis. Interestingly, it was also shown that a bimodal distribution of high- and low-affinity InsC-ChgA-specific CD4^+^ T cells exist in the islets of diabetic NOD mice, suggesting that two functionally different populations of these cells may contribute to disease in different ways (162). For example, high-affinity cells may be more likely to initiate disease pathology and epitope spreading while low-affinity cells may help maintain a long-term proinflammatory state later in disease (163). Regardless of the precise mechanism of hybrid peptide-specific CD4^+^ T cell antigen encounter, these cells are prime suspects for critical mediators of disease progression.

Hybrid peptide antigens are not limited to only murine InsC-ChgA and InsC-IAPP, as multiple hybrid peptides derived from various islet peptide fusion partners have been observed in both NOD mice and diabetic patients (151, 152, 154, 155). For example, both InsC-ChgA- and InsC-IAPP-specific CD4^+^ T cells have been identified in peripheral blood of patients with T1D (151, 154). Insulin-insulin hybrid peptides have also been frequently found, with InsC-InsC and InsC-InsA hybrids being the most common in diabetic patients relative to healthy controls (151, 154, 155). InsB left-half hybrid peptides have also been identified. InsB-neuropeptide Y-specific CD4^+^ T cells found within the islets of a diabetic patient secrete IFNγ upon *in vitro* stimulation, suggesting that these cells may be pathogenic effectors late in the disease process (152). Additionally, peripheral blood InsB:secretogranin I-specific CD4^+^ T cells have been identified in diabetic patients that possess a CD45RA^-^CCR7^-^ effector memory phenotype, implicating this hybrid peptide fusion product as a potential contributor to the development of a long-term autoimmune response (155). Examples of hybrid peptide fusions to InsB are particularly interesting because they suggest a mechanism whereby InsB_9-23_ may be cleaved at amino acid 21 to allow formation of a fusion protein that would essentially accomplish a R22E or R22D amino acid substitution required for InsB_9-23_ to favorably bind to diabetes-susceptibility MHC molecules in register 3 (82). Given the sizeable contribution of hybrid peptides to the total peptide pool in NOD mouse islets, it is likely that additional roles for (and different antigen specificities of) hybrid peptide-specific T cells will continue to emerge as the field progresses (164).

Although hybrid peptide fusion products are relevant to T1D pathogenesis, their mechanism of production is unknown (151–155). Fusion of disparate protein segments into hybrid peptides may proceed through a transpeptidation reaction known to occur in other physiological settings (165–167). This transpeptidation process would be expected to occur optimally under conditions supporting exceedingly high protein concentrations, such as within β-cell dense-core insulin secretory granules that contain over 200,000 insulin molecules alone per secretory granule (168). Supporting this idea, β-cells from diabetic patients express peptide:HLA-I complexes loaded with several different transpeptidation products (169). While it has been shown that cathepsin L can mediate the transpeptidation of insulin C-chain (InsC) and chromogranin A (ChgA) peptide fragments to form an InsC-ChgA hybrid peptide that is highly stimulatory to the diabetogenic murine BDC-2.5 CD4^+^ T cell clone, this cathepsin subtype is not normally found at high levels within pancreatic islets (170–172). This implicates other β-cell secretory granule enzymes as putative key mediators of the transpeptidation reaction.

### 2.9 Alternative neoantigen products

Importantly, PTMs other than hybrid peptides are also likely relevant to T1D pathogenesis, as deamidation, transglutamination, and citrullination of critical pathogenic epitopes recognized by CD4^+^ and CD8^+^ T cells have all been observed (151, 173, 174). Another class of neoantigens that may contribute to disease progression includes defective ribosomal products, a key example of which is an alternative open reading frame within human insulin mRNA recognized by CD8^+^ T cells capable of directly killing β-cells (175, 176). Additionally, mRNA splice variants may function as T cell neoantigenic targets given that 35% of human islet genes undergo alternative splicing, and splice patterns are altered following exposure to the proinflammatory cytokines IL-1β and IFNγ (177). Indeed, splice variants of IA-2 are uniquely expressed in islets relative to the thymus and spleen, and β-cells from diabetic patients express peptide:HLA-I complexes loaded with alternative mRNA splice products of preproinsulin and secretogranin V (169, 178). Although many examples of diabetes-related neoantigens have been identified, the role of each class in stimulating diabetogenic T cell responses is still unclear and requires further investigation.

### 2.10 Epitope spreading

Once pancreatic islets become inflamed and β-cell destruction begins, additional antigen exposure occurs through subsequent activation of more diverse antigen-specific T cell responses in a process termed epitope spreading (179, 180). Because CD4^+^ and CD8^+^ T cell numbers within pancreatic islets increase as disease progresses in diabetic patients, it is likely that epitope spreading occurs early in disease and rapidly accelerates thereafter (58, 181, 182). T cells from patients with T1D target a greater variety of epitopes from GAD65 and proinsulin proteins compared to partially HLA-matched healthy controls, demonstrating intra-protein epitope spreading in disease (179). Additionally, PBMC responses from autoantibody-positive individuals target an increasing number of islet proteins throughout time, suggesting that a temporal hierarchy of pathogenic epitopes may exist both within and between different β-cell-derived proteins (180).

Supporting a model of antigenic hierarchy, insulin appears to be an earlier target of antigen-specific immune responses given that tetramer staining of recent-onset diabetic patient islets shows mostly insulin-specific CD8^+^ T cells, whereas responses against IGRP, IA-2, GAD65, IAPP and preproinsulin are present in islets from patients with longstanding disease (58). Similarly, InsB_9-23_-coupled splenocytes induce tolerance in young NOD mice, but tolerance is not achieved using epitopes derived from IGRP or GAD (183, 184). In contrast, T_H_1 CD4^+^ T cell responses have been shown to target GAD_509-528_ and GAD_524-543_ before spreading to insulin in NOD mice, though this may simply be due to a lack of targeting the pathogenic InsB_9-23_ epitope (96). Proinsulin-specific T cell responses appear to be a prerequisite for IGRP-specific responses, since proinsulin-tolerant NOD mice do not develop IGRP-specific CD8^+^ T cell expansion even though IGRP-tolerant NOD mice develop insulitis and AD (122).

Taken together, it is apparent that a complex interplay between T cell responses of multiple antigen specificities evolves as disease proceeds, though the details of these relationships and their origins during the earliest phases of diabetes initiation require further inquiry. Future studies in mice that can target the endogenous polyclonal T cell populations selective to each autoantigens will help define which antigen(s) determine disease initiation and progression. Given that hybrid peptides are neoantigens formed in the periphery, we posit that hybrid peptide-specific T cells are the principal early drivers of autoimmune diabetes.

## 3 Presentation of autoantigen to T cells

### 3.1 Dendritic cells

Activation of self-reactive T cells in the context of T1D is potentially driven by the antigen presenting activity of several APC subtypes including dendritic cells (DCs), macrophages, B cells and islet β-cells mediated through loading self-antigen on MHC-I or MHC-II surface molecules. DCs represent particularly compelling candidates given their increased presence in the islets of T1D patients as disease progresses and their production of the proinflammatory cytokines TNFα and IL-1β (185). This increase in islet-localized DCs throughout time is likely mediated at least partially through the attractive actions of chemotactic cytokines released by islet macrophages, given that macrophage depletion in NOD mice prevents the accumulation of islet DCs (186). However, the presence of DC precursors with proliferative potential in fetal NOD pancreata suggests a possible inherent residence and self-renewal capacity as well (187).

Irrespective of the origin of pancreatic DCs, islet DCs have been shown to phagocytose dense-core, insulin-like granules and produce β-cell protein-derived peptide:MHC complexes, with similar DCs also appearing in the pLN (188, 189). Further, NOD islet DCs pulsed with β-cell secretory granules are capable of direct presentation of insulin peptides, and this process is dependent upon the intrinsic affinity of I-A^g7^ for pathogenic insulin peptides in both type 1 (XCR1^+^) and type 2 (SIRPα^+^) conventional DCs (cDC1s and cDC2s) (79, 190). This process is thought to be facilitated by widespread β-cell death early in life in NOD mice that increases β-cell antigen loading by CD11b^+^CD11c^+^CD8α^-^ DCs that subsequently traffic to the pLN for antigen presentation (191). Importantly, cDC1s are required for the development of autoimmune diabetes given that NOD.*Batf3^-/-^* mice do not develop islet immune cell infiltration or diabetes (*batf3* plays a central role in the development of conventional DCs) (192). Interestingly, adult NOD.*Batf3^-/-^* splenocytes transfer disease to NOD.*Rag1^-/-^* mice but not as efficiently as diabetic NOD splenocytes, suggesting a partial decrease in the pathogenic capacity of cDC1-deprived NOD T cells. This decrease in pathogenic capacity may be due to decreased CD8^+^ T cell effectors, because CD8^+^XCR1^+^BATF3^+^ cDC1s are also professional cross-presenting cells and thus also responsible for activation of diabetogenic CD8^+^ T cells (193, 194). These studies together support a model whereby islet cDC1s acquire antigen within the intra-islet space and subsequently traffic to the draining lymph node to induce activation of autoreactive T cells.

### 3.2 Macrophages

A role for macrophages in the pathogenesis of autoimmune diabetes has long been suspected given that macrophage depletion in NOD mice prevents disease, though the extent to which their diabetogenic properties are due to T cell-directed antigen presentation has been difficult to elucidate (186, 195, 196). Similar to islet DCs, resident islet macrophages produce TNFα and IL-1β; however, unlike DCs, there is not a need for replenishment by circulating monocytes, given that parabiosis experiments have demonstrated that islet macrophages undergo self-renewing proliferation (185, 197). Their location embedded within pancreatic islets permits CX_3_CR1^+^F4/80^+^ macrophages to regularly sample antigen derived from both intravascular sources in adjacent blood vessels as well as dense core secretory granules directly within β-cells, offering a mechanism by which macrophages might acquire auto-antigen for presentation (198). Additionally, it has been shown that macrophages are capable of insulin uptake from β-cell-derived secretory granules, a subset of which are likely obtained by sampling exosomes released by β-cells (189, 190). Using a model of macrophage depletion through administration of liposomal dichloromethyl diphosphonate to NOD.NY8.3 mice, insulitis and autoimmune diabetes were prevented and splenocytes were found to downregulate FasL and perforin, suggesting a role for macrophages in activating peripheral CD8^+^ T cells (121). Despite the evidence supporting macrophage antigen presentation as important for disease, it is crucial to note that macrophage depletion in NOD mice at late-stage prediabetic timepoints is just as effective at preventing disease onset as intervention at 3 weeks of age (186). Given that many autoreactive T cells have likely already become activated at late-stage diabetic timepoints, this result implies that macrophages exert the brunt of their pathogenicity through mechanisms distinct from direct antigen presentation such as expression of pro-inflammatory cytokine and nitric oxide release that results in direct β-cell damage, as well as enhancing further T cell recruitment once an autoimmune attack is underway.

One important mechanism by which tissue-resident macrophages may mediate β-cell destruction is via the local production inflammatory cytokines. Macrophages produce several inflammatory mediators upon viral infection and following recognition of other danger-associated molecular patterns (DAMPs), including IL-1β, TNFα, nitric oxide, and prostaglandins (22, 199, 200). Surprisingly, macrophage expression of inflammatory genes in response to diabetogenic viral infections is dependent on activation of the chemokine receptor CCR5 and not controlled by viral dsRNA sensors, suggesting novel and distinct signaling pathways in macrophages that may be implicated in β-cell damage (22, 201, 202). *In vitro* studies using isolated islets and β-cell lines show the activated macrophages inhibit insulin secretion, stimulate ER stress, cause DNA damage, and can result in β-cell death in a manner dependent on intra-islet macrophage cytokine release (203–209). Specifically, macrophage expression of IL-1β stimulates β-cell production of micromolar levels of nitric oxide, which results in these potentially deleterious (but reversible) effects on β-cell function and viability (203–209). Indeed, selective pharmacological blockade, antibody neutralization, or genetic deletion of inflammatory mediators such as IL-1β and inducible nitric oxide synthase (iNOS; the enzyme in β-cells responsible for nitric oxide production) can attenuate or prevent diabetes in response to encephalomyocarditis virus infection in genetically susceptible mice (210, 211), Kilham rat virus infection in the Bio-Breeding rat (20, 212–214), spontaneous diabetes in the NOD mouse (121, 215), and in other models of diabetes (216–218).

### 3.3 B cells

B cells are found in inflamed islets and are critical for autoimmune diabetes given that B cell-depleted NOD mice do not develop spontaneous disease (219–222). B cell self-antigen specificity is likely fundamental to influencing disease trajectory since B cells are not only known to bind the diabetogenic InsB_9-23_ epitope, but NOD mice also develop accelerated disease with V_H_ transgene insertion forcing insulin specificity in 1–3% of mature B cells (190, 223). At least a portion of B cell disease contributions are governed by β-cell autoantigen capture by the B cell receptor (BCR) given that transgenic BCR fixation to target a hen egg lysozyme (HEL) epitope on a B cell-depleted NOD background (NOD.*IgHEL.Ig*μ*^null^* mice) prevents stimulation of GAD-specific T cell responses *in vitro* despite adequate stimulation in the presence of only native B cells as APCs (224). Further, NOD.*IgHEL.Ig*μ*^null^* mice develop delayed onset of diabetes similar to NOD.*Ig*μ*^null^* mice, suggesting that B cell antigen presentation may actively promote disease progression (224). However, antigen identity is critical as artificial presentation of InsC-ChgA peptide on either MHC class I- or class II-expressing B cells prevents disease in a NOD.*scid* model of diabetes (225).

In support of a pathogenic role for B cell antigen presentation, islet-infiltrating B cells have enhanced expression of CD80 and CD86, and CD80/86 blockade to impair co-stimulation of T cells prevents *in vitro* NOD T cell proliferation when cultured with B cells as APCs (226). This study also demonstrated that co-transfer of diabetic NOD splenocytes with BCR-stimulated B cells to NOD.*scid* mice induces disease, yet disease can be nearly prevented when B cells are first incubated with anti-CD80 and anti-CD86 monoclonal antibodies (226). Importantly, NOD mice engineered with a deficiency in I-A^g7^ expression confined to the B cell compartment are protected from disease but still develop peri-insulitis, demonstrating that antigen presentation by B cells to CD4^+^ T cells is crucial for disease development, yet other APCs likely also contribute (227). A role for B cell activation of CD8^+^ T cells has also been suggested since B cell-deficient NOD.*Ig*μ*^null^* mice reconstituted with NOD B cells expectedly develop disease, but reconstitution with B cells from NOD.*β_2_m^-/-^* mice does not induce diabetes (β2 microglobulin is an essential component of MHC-I class molecules) (228). Taken together, these results strongly implicate B cells as important conduits for antigen presentation to both CD4^+^ and CD8^+^ T cells.

### 3.4 β-cells

Although it is generally thought that the primary role for β-cells in T1D pathogenesis involves functioning as a source of antigen for presentation by other cell types, evidence has emerged over time suggesting an additional role in direct antigen presentation as well (190, 229, 230). It is known that human β-cells express MHC-I, and β-cell class I peptidomes express peptide fragments derived from β-cell proteins (169, 229). Additionally, elevated glucose concentrations increase β-cell preproinsulin antigen presentation and death by cytotoxic T cells *in vitro*, unsurprisingly supporting the assertion that β-cells present antigen directly to cytotoxic CD8^+^ T cells (231). It has also been shown that deceased autoimmune diabetic children express HLA-DR in islets containing only insulin-producing cells, suggesting that β-cells are an islet endocrine cell type that uniquely expresses MHC-II (230). Notably, human β-cells also express class II transactivator mRNA whose protein product increases MHC-II levels, and its expression increases as islet infiltration proceeds (232). β-cell expression of MHC-II has also been demonstrated in the β-cells of infiltrated islets of transgenic NOD.NY4.1 (specific for an unknown islet antigen) mice; these MHC-II-expressing β-cells independently induce proliferation of diabetogenic BDC-2.5 CD4^+^ T cells *in vitro* (233). MHC-II expression in β-cells is context-specific, with proinflammatory cytokines (e.g. IFNγ and TNFα) capable of promoting MHC-II expression in human islets (233, 234). However, it remains controversial whether wild type NOD mice express MHC-II and whether MHC-II expression on human β-cells occurs in healthy conditions or only following cytokine exposure.

Given that expression of CD80 and CD86 is required for T cell activation during concurrent antigen presentation, it is surprising that no studies have demonstrated isolated expression of these molecules to β-cells (235, 236). Despite this, it has been shown that transgenic expression of CD80 in β-cells of C57BL/6 x NOD mice or C57BL/6 x DQ8^+^/mII^-^ (global expression of DQ8 without murine MHC-II) induces rapid development of autoimmune diabetes, suggesting that β-cells are indeed capable of direct antigen presentation (237, 238). Transgenic β-cell expression of CD80 does not require CD4^+^ T cell participation for β-cell destruction as diabetes-susceptible mice engineered to express the co-stimulatory molecule CD80 under control of the rat insulin promoter (RIP) within pancreatic β-cells (C57BL/6.RIP-B7.1 mice) develop disease in the absence of human or murine MHC-II, emphasizing the limitations of these artificial models (239). These studies in combination are challenging to interpret given the lack of evidence for an *in vivo* role of β-cell antigen presentation and the use of contrived murine study systems. Because of this, direct antigen presentation by β-cells, if relevant, likely comprises only a subtle portion of antigen presentation events and likely are responsible for direct cytotoxic T cell killing after T cell autoreactivity has already been firmly established.

## 4 Antigen-specific therapies in autoimmune diabetes

### 4.1 Unmodified antigen

The discovery of multiple diabetes-relevant antigens throughout time has laid the groundwork for the design of disease mitigating therapeutics targeting antigen-specific immune responses (summarized in **Table 1**). The simplest therapeutic strategy studied involves introduction of antigen to induce tolerance. Clinical trials evaluating full-length insulin protein administered to humans either subcutaneously (240), intranasally (241), or orally (242) found there to be no effect on delaying or preventing T1D onset. Despite the failure of this protein immunization strategy, peptide-based approaches have had more favorable outcomes. For example, administration of InsB_9-23_ or an altered peptide ligand version termed NBI-6024 to NOD mice significantly delays disease (84, 243), though no effect on islet autoantibodies or C-peptide levels is observed when NBI-6024 is given subcutaneously to diabetic patients (244). However, both proinsulin C19-A3 peptide (245) and GAD65-alum treatment (246–248) given to recently diagnosed T1D patients maintain C-peptide levels over time with variable success. The mechanism of peptide therapy protection appears to be in part T_reg_-mediated given that treatment of NOD mice and *in vitro* prediabetic human CD4^+^ T cells with InsB_9-23_ and proinsulin peptide promotes conversion of naïve T cells to FoxP3^+^ T_regs_ (84, 245, 249). Protein/peptide approaches can be augmented through coupling of toxins to diabetes-relevant antigens, with oral and nasal administration of cholera toxin B-coupled insulin reducing islet inflammation and disease onset in NOD mice through a mechanism that involves downregulation of CD86 and increased IL-10 expression by DCs (250–253). Saporin-coupled IGRP-specific MHC-I tetramers can delete IGRP-reactive CD8^+^ T cells and delay autoimmune diabetes as well (254).

**Table 1:** Key findings and potential interpretations for antigen-specific therapies in the treatment and prevention of autoimmune diabetes.

Unmodified antigen	• Whole protein administration in humans – no effect • Peptide-based approaches in mice and humans – variable success • Animal based studies suggest coupling antigen expression with immunosuppressive genes afford a more potent effect
Nano- and microparticles	• Antigen-coupled particles can tolerize mice to autoantigen and suppress disease development
Cellular approaches	• Antigen-coupled antibodies or cells can stimulate tolerance or deletion of autoreactive T cells in mice
CAR T cell therapy	• Engineered CAR Treg cells offer superior suppression and disease attenuation to polyclonal Treg cell treatments in animal models • CAR T cells now being used in other autoimmune diseases to selectively kill target autoreactive T cell populations • Each of these methods are novel and yet relatively unexplored in human clinical trials
Antibodies targeting Peptide:MHC	• Early data in mice showing inhibition of antigen presentation and decreased islet infiltration suggests this may be a potential therapy only during early disease course

DNA delivery techniques have also been employed, with a proinsulin plasmid improving C-peptide levels and decreasing proinsulin-specific CD8^+^ T cell numbers in diabetic patients (255). Plasmid therapies have been enhanced by encoding diabetogenic epitopes with immunosuppressive genes in one or more DNA vectors, as shown in studies combining GAD65 with IL-10 or the tolerogenic apoptosis-inducing molecule BAX to suppress autoimmune diabetes in NOD mice (256, 257). Extending this concept, other groups discovered that encoding multiple epitopes in one or more plasmids augments diabetes protection in NOD mice. For example, diabetes is suppressed with administration of a plasmid encoding ChgA, IGRP, GAD65 and insulin antigens, and targeting DβH_233-241_, ZnT8_158-166_, ZnT8_282-290_ and proinsulin with a plasmid approach provides better disease protection than proinsulin alone (258, 259). Another method of tolerogenic antigen administration involves oral delivery of *Lactococcus lactis* bacteria expressing IL-10 and proinsulin or GAD65 with simultaneous intravenous injection of αCD3 mAbs (monoclonal antibodies) to recently diabetic NOD mice (260–262). These therapies result in reversal of disease accompanied by increased FoxP3^+^ T_regs_ in the pancreas, blood, spleen, and pLN with suppressive activity dependent upon CTLA-4 and TGFβ signaling.

### 4.2 Nanoparticles and microparticles

Antigen-coupled nanoparticles (NPs) and microparticles (MPs) have also been explored as a possible tolerogenic therapeutic approach. Both insulin coupled to poly(lactic-*co*-glycolic acid) (PLGA) MPs and proinsulin coupled to gold methoxypolyethylene glycol-SH NPs prevent autoimmune diabetes when administered to prediabetic NOD mice, with mechanisms dominated by PD-1 upregulation on CD4^+^ and CD8^+^ T cells and increased splenic and pLN T_regs_ respectively (263, 264). Proinsulin peptide coupled to gold NPs injected into human breast skin samples are taken up by Langerhans cells and reduce antigen presentation, suggesting that induction of DC tolerance may contribute to proinsulin/insulin-coupled NP and MP disease inhibition (265).

Antigens other than insulin also demonstrate NP and MP therapeutic potential. For example, IGRP peptide-MHC-coupled iron oxide NPs prevent autoimmune diabetes in NOD mice by inducing suppression and killing of APCs and expansion of autoregulatory CD4^+^ and CD8^+^ T cells, and IGRP peptide-coupled PLGA NPs suppress disease in NOD.*scid* recipients of diabetogenic transgenic NY8.3 CD8^+^ T cells (266–268). Similarly, disease is inhibited in NOD.*scid* recipients following transfer of diabetogenic transgenic BDC-2.5 CD4^+^ T cells when mice are given p31- or InsC-ChgA-coupled PLGA NPs, and this result is recapitulated by co-treatment with p31-coupled acetylated dextran MPs and rapamycin (268–270). Importantly, spontaneous disease in NOD mice is delayed by subcutaneous co-injection of liposomal particles encapsulating a BDC-2.5 mimotope and vitamin D_3_, and disease is prevented with administration of BDC-2.5 mimotope-coupled iron oxide NPs (267, 271). Much like IGRP-specific NP and MP therapy, InsC-ChgA-specific treatments induce increased T_reg_ numbers and a decrease in T_H_1-like effector cells as contributory mechanisms to disease suppression (267, 269–271). Given these consistent results in achieving disease prevention with various diabetes-relevant antigens independent of the specific NP or MP coupling formulation employed, successful translation of this technology to human T1D may be possible with prudent selection of autoantigen targets to maintain minimal toxicity.

### 4.3 Cellular approaches

Cell-based strategies are additional methodologies for autoimmune diabetes therapy. Similar to antigen-coupled NP and MP approaches, 1-Ethyl-3-(3-dimethylaminopropyl)carbodiimide (ECDI) coupling of intact insulin or InsB_9-23_ to splenocytes and sortase-mediated coupling of InsB_9-23_ to red blood cells (RBCs) both generate tolerogenic apoptotic coupled cells capable of preventing diabetes in NOD mice (184, 272). Beyond direct chemical crosslinking of antigen to apoptotic splenocytes or RBCs, p31 can be targeted to RBCs using antigen constructs specific for the RBC surface marker glycophorin A to completely prevent BDC-2.5-transferred disease in NOD mice (273).

To directly target APCs for tolerance induction, antigen delivery to CD205^+^CD8^+^ DCs using αCD205 antibodies coupled to antigen has been performed and shown to promote antigen internalization (274, 275). This model has shown promise as a tolerogenic therapy given its consistent conversion of naïve T cells to T_regs_ through enhanced TGFβ secretion by CD205^+^ DCs across multiple experimental systems (276–278). Antigen-coupled αCD205 antibodies have found variable success across multiple diabetes-relevant antigens in the NOD mouse model: 1) Co-transfer of insulin mimotope-αCD205 and insulin-specific AI4 CD8^+^ T cells causes antigen-specific T cell deletion, and spontaneous disease is suppressed with proinsulin-αCD205 administration (279, 280); 2) Co-transfer of p63-αCD205 and transgenic BDC-2.5 CD4^+^ T cells enhances T_reg_ conversion despite not affecting disease incidence (280); and 3) IGRP_206-214_-αCD205 reduces the number of endogenous IGRP-specific CD8^+^ T cells and promotes deletion of transgenic NY8.3 CD8^+^ T cells in an adoptive transfer system (281). Interestingly, despite a lack of disease prevention with p63-αCD205 and transgenic BDC-2.5 CD4^+^ T cell co-transfer, p63-αDCIR2 targeting CD11b^+^ cDC2s could suppress BDC-2.5 CD4^+^ T cell-mediated disease, implicating multiple DC populations presenting various antigens as potential therapeutic foci (280, 282). While use of DC antigen-specific therapies in clinic has only recently been explored for T1D one study found that proinsulin pulsed tolerogenic DCs injected intradermally did not affect HbA1c, C peptide, hypoglycemic events or insulin dose required for symptom maintenance (283). However, the treatment was safe and caused only minor injection site symptoms, paving the way for future DC-based clinical applications (283).

### 4.4 *Ex vivo* and chimeric antigen receptor T_reg_

Work in animal models of autoimmunity have demonstrated the potent therapeutic potential that antigen-specific T-regulatory cells may have on diabetes prevention. The use of T_regs_ are appealing not only because of their specificity to auto-antigens, but because they are also capable of bystander (antigen-independent) suppression of other, local, autoreactive T-cells (284). Expanded transgenic BDC-2.5 T_regs_ were shown to attenuate BDC2.5 T-cell transfer-induced diabetes in NOD.scid mice (285, 286), demonstrating the feasibility of *ex vivo* T_reg_ expansion in inhibiting effector T-cell populations that share similar specificities. These same expanded BDC-2.5 T_regs_ were also capable of attenuating spontaneous diabetes in the following diabetes models: NOD.CD28^-/-^ mice (285), NOD.scid mice that were treated with splenocytes from diabetic NOD mice (286), or spontaneous diabetes in wild-type, pre-diabetic (13 weeks age) NOD mice (287). Importantly, BDC-2.5 T_regs_ inhibit disease in T_reg_-deficient NOD.*CD28^-/-^* mice better than anti-CD3ϵ-coupled bead-expanded polyclonal T_regs_, suggesting that T_regs_ specific for diabetogenic antigens may be clinically applicable and much more potent than polyclonal Tregs (288). Besides treatment with expanded antigen-specific T_regs_, chimeric antigen receptor (CAR) T_regs_ have recently been explored as a potential autoimmune diabetes therapy with variable success. Long-lived murine CAR T_regs_ expressing an insulin-specific single-chain variable fragment (scFv) proliferate and generate IL-2 upon *in vitro* stimulation and suppress proliferation of allogeneic CD8^+^ T cells, but have no effect on diabetes progression in NOD mice (289). These results highlight the robust bystander suppressive effect that T_reg_ cells of single islet-derived antigen specificity may have on limiting autoimmune progression even when autoreactivity to numerous epitopes has occurred. Additionally, these data may illustrate why, to date, all clinical trials that used expanded polyclonal T_reg_ cells have only demonstrated safety but not efficacy for disease prevention (290).

A number of limitations exist with *ex vivo* conversion and expansion of patient auto-reactive T-cells into T_reg_ cells that can then be autologously transplanted. Relevant autoreactive T cell populations are rare in peripheral circulation, and there may be wide variability in TCR affinity and specificity, limiting therapeutic potential. Two alternative methods can be conducted that could confer greater specificity, activation potential, and manipulability as opposed to expanding T cells with antigen followed by enrichment of antigen-specific T cells. First, T cells may be engineered to become T_reg_ cells with the desired antigen specificity (engT_regs_) by combining genetic editing to force express FOXP3 with lentiviral transduction of a specified TCR. The therapeutic potential of this approach was recently demonstrated by using BDC2.5^+^ engT_reg_ cells to prevent diabetes pathogenesis in mice receiving either BDC2.5^+^ T cells which demonstrated antigen-specific repression, or diabetic NOD splenocytes which demonstrated bystander suppressive capacity (291). The second approach is to develop chimeric antigen receptors (CARs) composed of an extracellular single-chain antigen-specific antibody (scFv) linked to modular intracellular co-stimulatory signaling domains. Our laboratory designed an scFv CAR T_reg_ specific to InsB chain peptide _10-23_ in the context of MHC-II; this CAR suppressed diabetes in NOD.RAG^-/-^ mice with co-transfer of BDC2.5 T cells and suppressed spontaneous diabetes in wild type NOD mice (292). These studies highlight the therapeutic potential for engineered and CAR T_reg_ cells therapy in the prevention of autoimmune diabetes in humans.

CAR T cell therapy has revolutionized cancer therapy by facilitating antigen-specific immune activation against tumor antigens (293). These same treatments that have demonstrated remarkable efficacy in clearing lymphomas and other tumors refractory to conventional cancer therapies are now being repurposed to prevent or cure autoimmunity by targeting pathogenic immune cell populations. For example, systemic lupus erythematosus (SLE) is a systemic autoimmune disease characterized by autoantibodies that mediate tissue damage; B cell depletion using rituximab, an anti-CD20 antibody is a common treatment used to treat SLE (294). However, SLE disease may be severe and refractory despite anti-CD20 depletion. CD8^+^ CAR T cells specific to CD19 were shown to effectively deplete B cells, reverse autoantibody production and tissue damage, and extend the life span of multiple murine models of lupus (295). Remarkably, initial clinical trials in humans have demonstrated that in patients with severe SLE refractory to conventional treatments, anti-CD19 CAR T cells were able to achieve complete remission in all 5 patients within 3 months of treatment, and these patients continue to be seronegative for autoantibodies and without symptoms for months after B cells repopulated (296). However, CAR T cell therapies that broadly target entire immune subsets, such as CD19 expressing B cells, results in broad immunosuppression risk. To circumvent this, CAR T cells are now being developed that selectively deplete autoantibody-expressing B cells. For example, NMDAR-specific chimeric autoantibody receptor (CAAR) T cells, comprised of NMDAR auto-antigen fused to CD8 hinge and intracellular signaling domains, were able to selectively kill anti-NMDAR cell lines *in vivo* (297). The selective targeting of autoreactive TCR populations is a compelling method to extrapolate to both mouse models of diabetes and genetically at-risk humans with the goal of disease prevention.

### 4.5 Peptide: MHC

Peptide: MHC-specific approaches represent another potential autoimmune diabetes therapy, first suggested by results demonstrating a delay in diabetes progression and induction of antigen-specific antibodies following immunization of 4-week-old NOD mice with recombinant InsB_12-22_ register 3 (I-A^g7^-B:RE#3) peptide (298). Extending this approach, an anti-InsB_12-22_RE#3:I-A^g7^ monoclonal antibody was found to delay autoimmune diabetes in NOD mice by broadly decreasing pancreatic islet infiltration of both CD4^+^ and CD8^+^ T cells (85). Additionally, an I-A^g7^-B:RE#3-specific CAR CD8^+^ T cell treatment administered to NOD mice homed well to the pLN and delayed disease after one infusion, suggesting that the mechanism of these diverse therapies relies upon inhibition of the antigen presenting capabilities of relevant APCs (299). However, the utility of this approach is not limited to the I-A^g7^-B:RE#3 antigen, as immunization of NOD mice at 4- and 8-weeks with an anti-InsC-ChgA:I-A^g7^ delays the onset of autoimmune diabetes as well (300). To improve the production of anti-peptide:MHC monoclonal antibodies, a novel method for peptide:MHC mAb generation using a magnetic enrichment protocol was developed and resulted in generation of an anti-p63:I-A^g7^ monoclonal antibody with exceptionally high affinity that could prevent tolerance induction in NOD mice following transfer of p63-coupled cells (301). Although disease prevention was not shown in this study, this methodology will likely be helpful for the future production of peptide:MHC-specific monoclonal antibodies (301). The use of such antigen-specific therapies in clinic has not yet gained momentum, but the success of the various approaches detailed here using *in vitro* and murine models offer compelling evidence that further refinement of these techniques may yield novel T1D therapies for humans.

## 5 Conclusions

T1D is a disease characterized by T cell-mediated destruction of the insulin-producing β-cells within the pancreatic islets of Langerhans, so determining the antigenic targets of these T cell responses is critical to understanding disease pathogenesis and devising therapeutic approaches. Several T cell islet autoantigens have been identified as key contributors to disease progression, including insulin, GAD, IGRP, ChgA and IAPP; hybrid peptide formation among these various epitopes may provide an additional essential mechanism by which central tolerance is bypassed during the early development of autoimmunity. DCs, macrophages, B cells and β-cells may all participate in islet self-antigen presentation to autoreactive T cells, though the precise sites of antigen acquisition and presentation have not been globally determined. Critically, islet antigen-specific T cells have been successfully targeted for autoimmune diabetes prophylaxis and therapy in NOD mice and humans using several treatment modalities.

Despite tremendous progress in understanding the roles of different antigen-specific T cell responses in autoimmune diabetes, several critical outstanding objectives remain. First, while HLA haplotype, other genetic risk factors, viral infection, and gut microbiome are all well described risk factors that lead to the induction of autoimmune diabetes, the exact mechanism by which each of these factors lead to emergence of a sustained autoreactive T cell response is still incompletely elucidated. Epitope discovery using methods such as mass spectrometry on size-eluted microbiome antigenic fractions found to stimulate pathogenic T cells is necessary to characterize the cross-reactive T cell responses that might occur between similar epitopes derived from non-self vs. pancreatic β-cell proteins. Second, it is necessary to further characterize the exact timing that each antigen-specific T cell develops as well as its related pathologic contribution in autoimmune diabetes. Determining the temporal and pathogenic hierarchy of antigen-specific T cell responses will assist in identifying important therapeutic targets for the amelioration of disease.

Third, additional effort is required to address differences in T cell antigenic targets between the NOD mouse and humans with autoimmune diabetes. This will be especially important for hybrid peptides given that T cell responses target alternate peptide fusion products in humans compared to NOD mice (151, 152, 154, 155), and different mechanisms of hybrid peptide formation may underly this disparity. Fourth, successful therapeutic strategies in NOD mice must be effectively translated to clinic. Although numerous antigen-specific treatments demonstrate success in NOD mice, many rely upon frequent dosing and/or therapeutic administration prior to symptom onset (84, 85, 243, 258, 263, 264, 280, 298, 300). Further, many clinical therapeutics administered to recent onset T1D patients demonstrate negligible impact on disease trajectory even with alterations in laboratory markers (244–248, 255, 283). To overcome these challenges, it is necessary to devise approaches that accurately identify likely antigen-specific T cell responses in at-risk patients. Further, therapies that reduce treatment frequency (e.g. CAR T_reg_ infusion) should be considered to enhance patient compliance and affordability.

Development of an accurate autoimmune diabetes pathogenesis model requires detailed understanding and characterization of the myriad antigen-specific T cell responses underlying disease. Although many unanswered questions remain, substantial progress has been made in elucidating the identity of key autoantigens and the mechanisms permitting recognition and activation of their cognate T cells. Given the remarkable success of antigen-specific treatments in NOD mice, it is not unreasonable to expect further therapeutic application of antigen-specific approaches in clinical settings in the future.
